# Estimation of the Knee Adduction Moment and Joint Contact Force during Daily Living Activities Using Inertial Motion Capture

**DOI:** 10.3390/s19071681

**Published:** 2019-04-09

**Authors:** Jason M. Konrath, Angelos Karatsidis, H. Martin Schepers, Giovanni Bellusci, Mark de Zee, Michael S. Andersen

**Affiliations:** 1 Department of Technology, Xsens Technologies B.V., Enschede 7521 PR, The Netherlands; angelos.karatsidis@xsens.com (A.K.); hmartinschepers@gmail.com (H.M.S.); giovanni.bellusci@xsens.com (G.B.); 2 Department of Biomedical Signals and Systems, Technical Medical Centre, University of Twente, Enschede 7500 AE, The Netherlands; 3 Department of Health Science and Technology, Aalborg University, Aalborg 9220, Denmark; mdz@hst.aau.dk; 4 Department of Materials and Production, Aalborg University, Aalborg 9220, Denmark; msa@mp.aau.dk

**Keywords:** IMU, knee osteoarthritis, wearable technology, motion capture, musculoskeletal model

## Abstract

Knee osteoarthritis is a major cause of pain and disability in the elderly population with many daily living activities being difficult to perform as a result of this disease. The present study aimed to estimate the knee adduction moment and tibiofemoral joint contact force during daily living activities using a musculoskeletal model with inertial motion capture derived kinematics in an elderly population. Eight elderly participants were instrumented with 17 inertial measurement units, as well as 53 opto-reflective markers affixed to anatomical landmarks. Participants performed stair ascent, stair descent, and sit-to-stand movements while both motion capture methods were synchronously recorded. A musculoskeletal model containing 39 degrees-of-freedom was used to estimate the knee adduction moment and tibiofemoral joint contact force. Strong to excellent Pearson correlation coefficients were found for the IMC-derived kinematics across the daily living tasks with root mean square errors (RMSE) between 3° and 7°. Furthermore, moderate to strong Pearson correlation coefficients were found in the knee adduction moment and tibiofemoral joint contact forces with RMSE between 0.006–0.014 body weight × body height and 0.4 to 1 body weights, respectively. These findings demonstrate that inertial motion capture may be used to estimate knee adduction moments and tibiofemoral contact forces with comparable accuracy to optical motion capture.

## 1. Introduction

Knee osteoarthritis (KOA) is a major cause of pain and disability in the elderly population, with sufferers having reduced functional mobility [1]. KOA normally affects the medial compartment of the tibiofemoral joint, with the loss of medial cartilage being a distinguishing factor in the severity of the disease [2,3]. In addition to this, the presence of bone marrow lesions in subchondral bone has found to be associated with progression of the disease [4,5]. Cartilage appears to respond to the applied loads they experience and it has been suggested that the mechanical environment of the knee during gait can influence the breakdown of articular cartilage [6]. Thus, characterizing the mechanical loading of the tibiofemoral joint during daily living activities in the participant’s natural environment, may provide valuable insight into understanding the initiation and development of KOA in the elderly.

One of the most difficult challenges experienced by those that suffer KOA is the ability to negotiate steps. Ambulation of stairways is one of many important parts of human lifestyle activities and whilst this activity is easy for healthy individuals, it may be very challenging for the elderly and individuals with lower limb pathologies [7]. Stairclimbing places greater demands on range of motion of the lower limb than level walking [8,9] and individuals with KOA tend to take longer to ascend and descend stairs [10], while also demonstrating altered gait strategies [11,12].

In vivo measurement of articular loading is only possible through the use of instrumented knee implants, however due to their invasiveness, and because the joint replacements alter the natural joint mechanics, these are not feasible to study healthy or OA joint mechanics. Previous studies have used the peak knee adduction moment (KAM) as a convenient surrogate for medial-lateral load distribution in those with KOA, which has been linked to the onset, progression and severity of disease [13,14]. Conventionally, the KAM is calculated via inverse dynamics using opto-reflective marker data in conjunction with force plate data in gait laboratories [15,16]. However, it is important to note that estimations of articular loading should also include the contribution of the surrounding muscles, since muscle forces are important contributors to the joint contact force [17]. Tibiofemoral joint contact forces (TJF) can be estimated using computational musculoskeletal models, which allow further analysis of muscle-tendon kinematics, muscle-tendon force and joint contact force calculations. Musculoskeletal analysis provides valuable information into understanding the way the body tissues are loaded, however their use is limited to the laboratory setting.

A limitation of laboratory-based methods of 3D motion capture is the availability and cost of laboratories, restricted measurement space and line-of-sight problems with markers [18]. Moreover, estimations during daily living activities outside of the laboratory are not possible. These shortcomings have led to the development of algorithms that allow the estimation of ground reaction forces and moments using exclusively kinematic data [19,20]. Inertial motion capture (IMC) enables the assessment of segment orientation and full body motion capture in laboratory free settings [21]. Importantly, IMC has been shown to estimate joint angles with good accuracy [22] and has been used to estimate 3D ground reaction forces and joint moments during gait, providing comparable accuracy with optical motion prediction [19]. However, no literature to date has used IMC to estimate the KAM and TJF during stair climbing and sit-to-stand activities in the elderly population, which have a high prevalence of developing KOA [23].

To this end, the aim of the present study was to use a musculoskeletal model to estimate the KAM and TJF during daily living activities in the elderly, using only IMC-derived kinematics. The estimated tibiofemoral moments and forces were synchronously validated using an equivalent musculoskeletal model with optical and force plate data as inputs. We hypothesized that a musculoskeletal model driven by IMC would provide estimates of tibiofemoral joint moments and contact forces, with comparable accuracy to musculoskeletal models driven by optical motion capture and force measurements. Such knowledge may enable applications that provide suitable estimates of tibiofemoral joint loading during daily living activities of the elderly in their natural environments.

## 2. Materials and Methods

### 2.1. Subjects

Eight healthy elderly participants (6 male, 2 female, 59 ± 8 years of age, body mass 79 ± 9 kg, height 175 ± 7cm) that met the inclusion criteria were recruited for the study. The inclusion criteria included the following (i) participants were aged between 50 and 75 years and (ii) participants had a body mass index (BMI) between 20 and 35. Exclusion criteria included the following (i) previous lower extremity surgery, (ii) swelling in one or both knees and (iii) inability to comply with the testing protocol. Measurements were performed at the Human Performance Laboratory, Department of Health Science and Technology, Aalborg University, Aalborg, Denmark. The experiment was carried out in accordance with the ethical guidelines of the North Denmark Region Committee on Health Research Ethics. Participants provided their written informed consent prior to data collection.

### 2.2. Experimental Protocol

**Figure 1:**
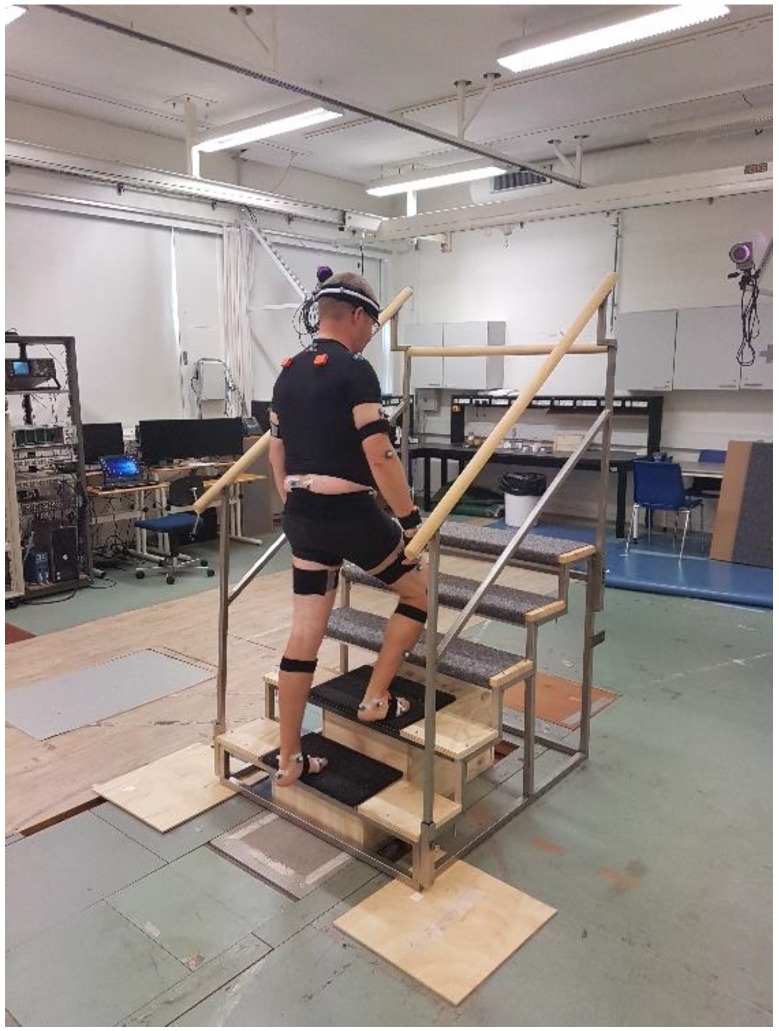
Participant undergoing a step up trial on a customized staircase. The first two steps are isolated from each other and the rest of the staircase and attached to the respective force plates.

Gait analysis was performed at the Human Performance Laboratory, Aalborg University, Aalborg, Denmark. Inertial motion capture was measured using an Xsens IMC system (Xsens Awinda, Xsens Technologies BV, Enschede, Netherlands) sampled at 60 Hz and processed by the matching software Xsens MVN Analyze 2018 (Xsens Awinda, Xsens Technologies BV, Enschede, Netherlands). 17 IMU modules were mounted on the head, sternum, pelvis, upper legs, lower legs, feet, shoulders, upper arms, forearms and hands. Opto-reflective motion capture was synchronously obtained using an 8-camera Qualysis (Oqus 300 series, Qualysis AB, Gothenburg, Sweden) motion capture system that sampled motion at 120 Hz. 53 Opto-reflective markers were affixed to the skin surface on the participants’ body segments atop prominent anatomical landmarks in accordance with a previously published full-body marker set [19]. The marker trajectories were filtered using a fourth-order, zero-phase Butterworth low-pass filter with a cut-off frequency of 6 Hz. In addition, ground reaction forces and moments were measured from three mechanical strain gauge force plates (Advanced Mechanical technology Inc., Watertown, MA, USA) sampling at 1200 Hz and time synchronized to the motion capture data. For the stair climbing trials, a special customized staircase was built that ensured that the first two steps were attached to two independent force plates and were isolated from each other, as well as the rest of the staircase (Figure 1).

Prior to performing trials, each participant stood in an upright posture while their segment dimensions were measured and subsequently entered into the Xsens MVN software. The measurements consisted of the distances of the ankle, knee, hip and top of head from the ground; pelvis and shoulder width; as well as the length of the foot. The IMC system was calibrated with the participant holding a neutral pose, followed by a walk calibration [24]. Xsens MVN 2018 then performs a sensor-to-segment calibration procedure that relates the 17 sensor orientations to derive the kinematics of 23 body segments [24]. Participants were then required to perform a static calibration trial recorded by the optical motion capture system, from which the opto-reflective markers identifying prominent anatomical landmarks were later used to scale and register the generic musculoskeletal model.

Once both motion capture systems were calibrated, the participants underwent 3D motion analysis. This involved a series of tasks, while the two motion capture systems concurrently and synchronously recorded 3D body motion and ground reaction forces and moments. The participants were instructed to perform at least four successful trials of each task; stair ascent, stair descent at self-selected speed and sit-to-stand. For the stair trials, a trial was deemed successful if the foot of the desired leg landed cleanly within the boundaries of the force plate/step. This meant that if the foot landed partially in contact with the ground, or another stair step, the trial was discarded. For the sit-to-stand trials, the right and left feet were placed on two separate force plates, while a third force plate that contained a wooden rectangular box was used for the participant to sit on and measure the corresponding reaction force of the chair. The participants begun seated with their feet placed shoulder width apart with their arms by their side, from which they were instructed to stand upright.

### 2.3. Experimental Procedures

All musculoskeletal models were constructed in AnyBody Modeling System (AMS) v.7.0 (Anybody Technology A/S, Aalborg, Denmark) [25], with the GaitFullBody template of the AnyBody Managed Model Repository (AMMR) 1.6.2 being used to reconstruct the musculoskeletal models. The lower limb model was derived from the Twente Lower Extremity Model [26], the model of the Delft Shoulder Group was used for the shoulder and upper limb [27], while the lumbar spine model was derived from the study of de Zee et al. [28]. The full body kinematic model contained a total of 39 degrees of freedom (DOF). More specifically, a pelvis segment with three rotational and three translational DOF, two spherical hip joints, two revolute knee joints, two universal ankle joints, three DOFs between pelvis and thorax, two gleno-humeral joints with five DOF each, two universal elbow joints and two universal wrist joints. Motions of the neck were locked in a neutral position.

For the models in which kinematics were determined by optical motion capture (OMC), the markers identifying prominent anatomical landmarks recorded from the static calibration trials were used to scale the generic musculoskeletal model. This was accomplished by optimizing the segment lengths and local marker coordinates of markers not located on bony landmarks until the least-square difference between modeled and measured marker trajectories was minimized. Once the models were scaled, the marker trajectories acquired from the dynamic trials were used to determine the joint angles for the scaled musculoskeletal models using inverse kinematics [29]. A muscle recruitment problem was solved by optimizing a system of equations that have been described in detail in a previous publication [30]. Briefly, the system of equations minimizes a cost function that is subject to dynamic equilibrium equations and non-negativity constraints, so that each muscle can only pull, but not push, while its force remains below its maximum isometric strength [25,31,32]. Inverse dynamics was performed, with the ground reaction forces and moments (GRF&M) used as inputs in combination with the 3D joint angles, to determine the knee adduction moment and joint reaction force during each of the daily living tasks.

For the models in which kinematics were determined by IMC and the GRF&M were predicted; a Biovision Hiearchy (BVH) file was exported from Xsens MVN Analyse 2018 into AMS where a stick-figure model was initially reconstructed. For each participant, their standing reference trial was used to identify their segment lengths and virtual marker positions, using the same least-square minimization between the model virtual markers and experimental markers [33]. Using the stick figure generated from Xsens MVN Analyze from measured body dimensions, the lengths of the shanks, thighs, head, upper arm and forearms were derived. The width of the pelvis, foot length and trunk height were optimized based on the aforementioned least squares least square minimization method [29]. Following this, the estimated segment lengths were used to perform inverse kinematic analysis on each of the dynamic trials, using the method of Andersen et al. [29]. This was accomplished by applying a virtual marker approach in which corresponding virtual markers on the stick-figure and the musculoskeletal model were defined and the least-square difference between these were minimized using the method of Andersen et al. [29]. The GRF&M were predicted using a modified method of Skals et al. [31]. 1 mm below the inferior surface of each foot, a set of 18 dynamic contact points were overlayed, each of which consisted of five unilateral force actuators. Each actuator could generate a positive vertical force perpendicular to the ground, with static friction forces in the anterior, posterior, medial and lateral directions using a friction coefficient of 0.5. The height and velocity activation thresholds were set at 0.03 m and 1.2 m/s respectively. Using these predicted GRF&Ms, inverse dynamics was performed to determine the knee adduction moment and tibiofemoral contact force.

### 2.4. Data Analysis

Data analysis focused on evaluating the performance of the IMC-driven musculoskeletal model in estimating the KAM and TJF, versus the OMC-driven musculoskeletal model. In addition to this, the kinematics of both methods of motion capture were compared. The root-mean-squared errors for each kinematic and kinetic variable were determined, while Pearson’s R-square correlation was used to quantify the agreement and consistency between the two estimation systems. Additionally, for the KAM and TJF, point to point estimates of agreement were established using a paired-sample T-test at each point of the stance phase [34], statistical significance was accepted for *p* < 0.05. Data analysis was performed in MATLAB 2017a.

## 3. Results

**Figure 2:**
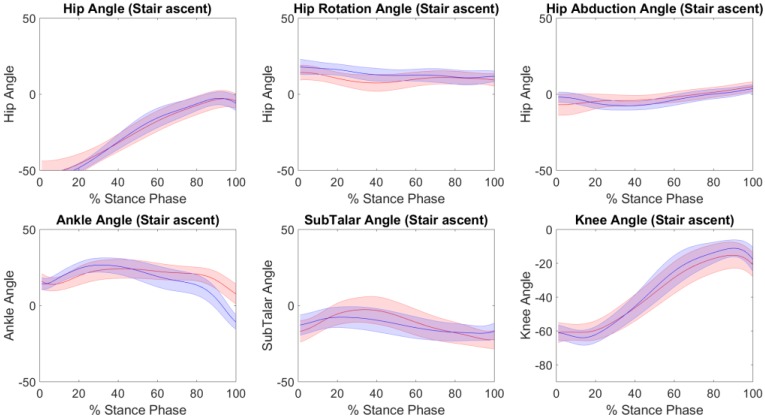
The kinematics (mean ± standard deviation) from the inertial motion capture (red) versus the optical motion capture (blue), across the stance phase for the stair ascent trials.

**Figure 3:**
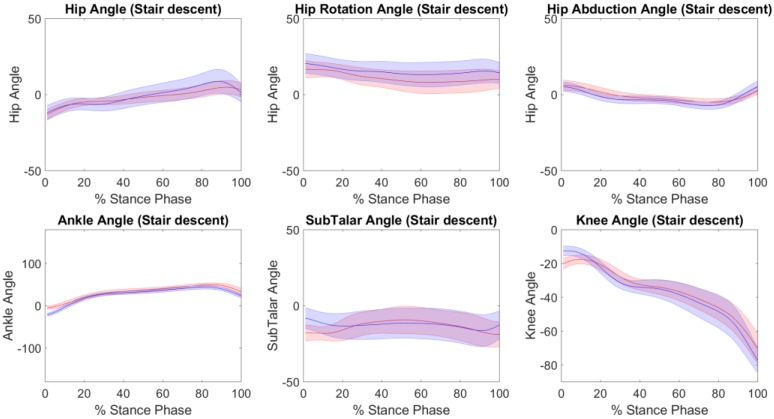
The kinematics (mean ± standard deviation) from the inertial motion capture (red) versus the optical motion capture (blue), across the stance phase for the stair descent trials.

**Figure 4:**
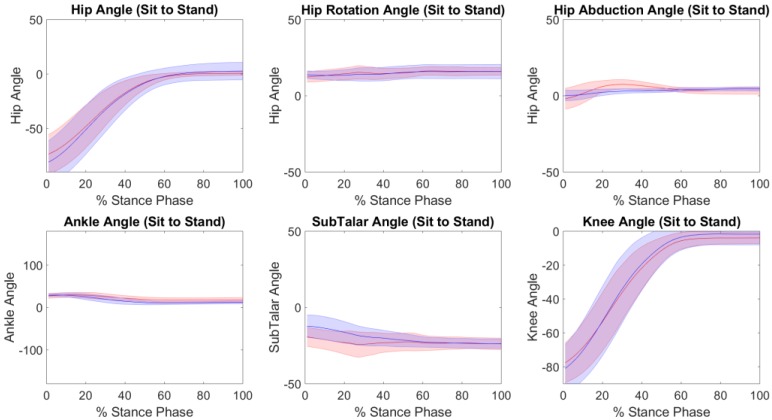
The kinematics (mean ± standard deviation) from the inertial motion capture (red) versus the optical motion capture (blue), across the stance phase for the sit to stand trials.

The accuracy of the kinematics, knee adduction moments and tibiofemoral contact forces predicted by IMC with respect to OMC are presented in Table 1 for each of the performed tasks. Across all tasks, excellent Pearson correlation coefficients were found in the sagittal plane kinematics, with RMSE’s of 4° to 7°, 3° to 4°, and 6° to 7° for the hip, knee and ankle respectively. Strong Pearson correlation coefficients were found for subtalar eversion, hip abduction and hip rotation across all tasks, with the exception of moderate and weak Pearson correlation coefficients for hip abduction in sit-to-stand trials. Across both tasks, RMSE’s of 5° to 8°, 2° to 3°, and 3° to 5° were found for subtalar eversion, hip abduction and hip rotation respectively (Table 1). Figure 2, Figure 3 and Figure 4 illustrate the kinematics averaged across stance phase for all participants, for the stair ascent, stair descent and sit to stand respectively.

**Figure 5:**
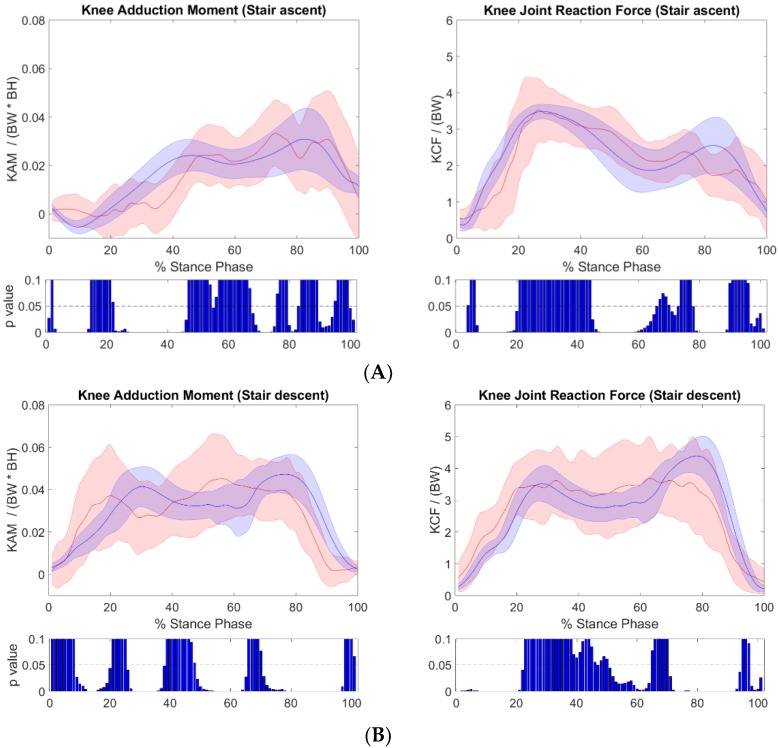

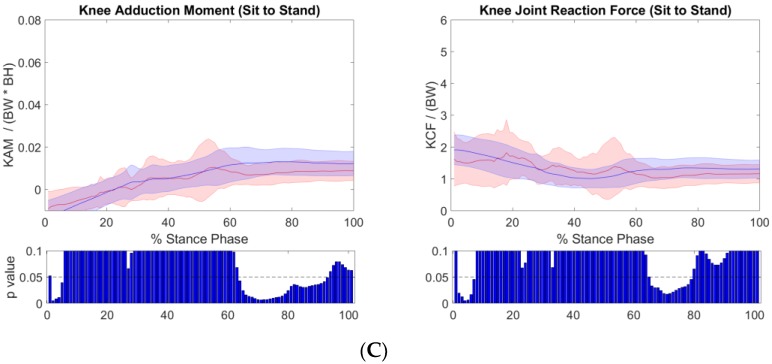
The Knee Adduction Moment and Knee Joint Reaction Force across the stance phase from the IMC-driven musculoskeletal model (red) versus the OMC-driven musculoskeletal model (blue), for each of the daily living tasks (**A**) stair ascent, (**B**) stair descent and (**C**) sit to stand. Point by point p-values are also shown for each point of the gait cycle.

Figure 5 illustrates the curves of the knee adduction moment normalized to bodyweight times height and the tibiofemoral contact force (knee joint reaction force) normalized to bodyweight, averaged across the stance phase for all participants. For the KAM, strong Pearson correlation coefficients were found for the stair ascent trials (r = 0.86), with RMSE’s of 0.01 ± 0.003 BW × BH (mean ± standard deviation). Stair descent trials showed strong Pearson correlation coefficients (r = 0.74), with RMSE’s of 0.014 ± 0.005 BW × BH. Excellent Pearson correlation coefficients (r = 0.98) were found for sit stand trials with an RMSE of 0.006 ± 0.002 BW × BH (Table 1).

For the tibiofemoral contact force normalized to bodyweight, and averaged across stance phase for all participants, strong Pearson correlation coefficients were found (r = 0.86) for stair ascent trials, with RMSE’s of 0.89 ± 0.32 BW (mean ± standard deviation). Stair descent trials showed strong Pearson correlation coefficients (r = 0.85), with RMSE’s of 0.9 ± 0.3 BW. Meanwhile, excellent Pearson correlation coefficients (r = 0.92) were found for sit-to-stand trials with an RMSE of 0.4 ± 0.14 BW (Table 1). The point-by-point assessment highlighted some portions of the stance phase in stair ascent and stair descent, in which deviations were more prominent. For sit-to-stand movements, no deviations were observed for the majority of stances (Figure 5).

## 4. Discussion

The aim of the present study was to inform a musculoskeletal model using only IMC-derived kinematics, in order to estimate the KAM and TJF in the elderly during daily living activities. The findings demonstrated that a musculoskeletal driven by IMC provide comparable estimates of TFJ moments and contact forces; with comparable accuracy to the same musculoskeletal model using optical motion capture and force plate measurements. Such knowledge may enable applications to analyze the knee joint function of elderly patients during daily living activities in their natural environment.

The accuracy of the kinematics predicted by IMC with respect to OMC showed excellent results during stair climbing and sit-to-stand activities, with strong to excellent Pearson correlation coefficients and RMSE’s between 2° and 8°. In support of this, Bergmann et al. [35] used IMU’s to assess anatomical joint angles during stair climbing and found strong correlations, as well as RMSE’s of 4 degrees over hip, knee and ankle joints. Furthermore, Karatsidis et al. [30] recently used IMC to inform a musculoskeletal model during gait at three different speeds and similarly reported excellent Pearson correlation coefficients with RMSE’s less than 6 degrees in the sagittal plane. To date, many studies have compared the use of portable IMU’s to track human kinematics with respect to optical motion capture and have found similar accuracies [19,24,36,37]. Importantly, segment positions and orientations are estimated via a sensor to segment calibration procedure, which applies the sensor orientation estimates to a scaled biomechanical model with the participant in a known pose [24]. Typically, an N-pose or T-pose is used for this sensor- to-segment calibration, however the assumption of a pre-determined pose may be partly violated and may lead to orientation errors that exceed 5 degrees [38]. In the present study, assistance was given to the participant to ensure they were positioned in the desired neutral posture during calibration.

In the present study, we were able to show that a musculoskeletal model driven by IMC in combination with a GRF&M prediction [31] provided reasonable estimates of the KAM during stair climbing and sit-to-stand activities. The KAM typically shows a characteristic double hump pattern during walking and stair climbing [39]. This characteristic double hump pattern can be observed in the present study, with the IMC-driven musculoskeletal model showing strong to excellent Pearson correlation coefficients with respect to the OMC MSK model; as well as displaying RMSE’s of 0.01 BW*BH and 0.016 BW*BH in stair ascending and descending respectively (Table 1). This may have important implications, as the KAM is often used as a convenient surrogate for medial-lateral load distribution in those that suffer from KOA and has also been linked to the onset, progression and severity of the disease [13,14].

To our knowledge, this study is the first to estimate tibiofemoral joint contact forces during stair climbing using only inertial measurement units. Overall, when compared to traditional methods using OMC and force plate data, we found that the IMC-driven musculoskeletal model showed strong Pearson correlation coefficients with RMSE’s of less than 1 bodyweight across all tasks. Using a musculoskeletal modelling approach, Taylor et al. [40] estimated the tibio-femoral joint contact force to be 5.4 bodyweights; while Costigan et al. [41] found an average force of 3 bodyweights, but also observed forces as high as 6 bodyweights. In recent times, to overcome uncertainties of mathematical models, telemetrized implants were developed to measure joint contact forces in vivo. Kutzner et al. [42] measured knee joint contact forces during various daily living activities and found that resultant forces lay typically in the range of 220% and 350% of the participant’s bodyweight. Similar values have also been reported in other in-vivo studies using instrumented implants [43,44,45]. The present study estimated approximately 2 bodyweights during sit to stand, which had the lowest contact forces of the tasks estimated; while approximately 4 bodyweights were measured during stair descent, which had the highest contact forces of the tasks estimated. However, it is important to note the differences in populations across studies, studies reporting instrumented implant data contain patients that underwent total knee replacement, while the present study contained healthy controls as the participants. Collectively, inertial motion capture shows great promise in terms of its accuracy with respect to optical methods. The portability of the system is a huge advantage, which may allow a wider adoption in the clinical community and allow more measurement sessions to be tracked over time.

The present study was not without its limitations. The kinematics measured with IMC may contain sensor to segment calibration errors due to a mismatch between the N or T-pose practiced, and what is modeled. This in turn could influence the estimation of the joint contact force due to differences in kinematics. Possible imperfections in scaling of the model may be another source of error that effects the accuracy. Furthermore, the stick figure model created contained more DOFs compared to the musculoskeletal model. Errors in the center of mass locations and inertial parameters of each human body segment may exist, which were calculated based on anthropometric tables in the literature. Finally, an inherent limitation to joint contact force estimation is the use of models to estimate joint loading, which may entail errors in both the IMC and OMC estimates.

## 5. Conclusions

This study investigated the accuracy of using a musculoskeletal model with IMC-derived kinematics to estimate the KAM and TJF in the elderly during typical daily living activities. The findings demonstrated that a musculoskeletal model driven by IMC can provide estimates of tibiofemoral joint moments and contact forces, with comparable accuracy to the same musculoskeletal model using optical motion capture and force plate measurements. Such knowledge may enable applications to analyze knee joint function of elderly patients during daily living activities in their natural environment. The proposed set-up is completely wearable and may now facilitate wider adoption in the clinical community.

## Figures and Tables

**Table 1:** Root mean squared error (RMSE) (mean ± standard deviation) and Pearson moment correlations (Corr) between inertial and optical motion capture for each task. Parameters include joint kinematics (degrees), knee adduction moment (bodyweight * bodyheight) and knee joint reaction force (bodyweights).

	Stair Up		Stair Down		Sit to Stand	
	Corr	RMSE	Corr	RMSE	Corr	RMSE
**Kinematics**						
**SubTalar Eversion**	0.87	8 ± 4	0.256	6 ± 3	0.97	5 ± 3
**Ankle Plantar/Dorsi Flexion**	0.78	6 ± 2	0.98	6 ± 2	0.97	7 ± 3
**Knee Flexion/Extension**	0.99	4 ± 3	0.99	3 ± 2	0.99	4 ± 3
**Hip Flexion/Extension**	0.99	6 ± 4	0.95	4 ± 2	0.99	6 ± 2
**Hip Abduction**	0.77	3 ± 1	0.91	2 ± 1	0.53	3 ± 1
**Hip Rotation**	0.51	5 ± 2	0.96	4 ± 2	0.68	3 ± 2
**Kinetics**						
**Knee Adduction Moment**	0.86	0.01 ± 0.003	0.74	0.014 ± 0.005	0.98	0.006 ± 0.002
**Knee Joint Reaction Force**	0.86	0.89 ± 0.32	0.85	0.9 ± 0.3	0.92	0.4 ± 0.14
